# Supplementary material for: Triple-Negative Breast Cancer Intrinsic FTSJ1 Favors Tumor Progression and Attenuates CD8+ T Cell Infiltration
Source: Cancers (Basel). 2024 Jan 31;16(3):597. doi: 10.3390/cancers16030597 (PMC10854779; doi:10.3390/cancers16030597)
Supplement: Supplementary file 1 [file cancers-16-00597-s001.zip › cancers-2825127-supplementary.pdf]

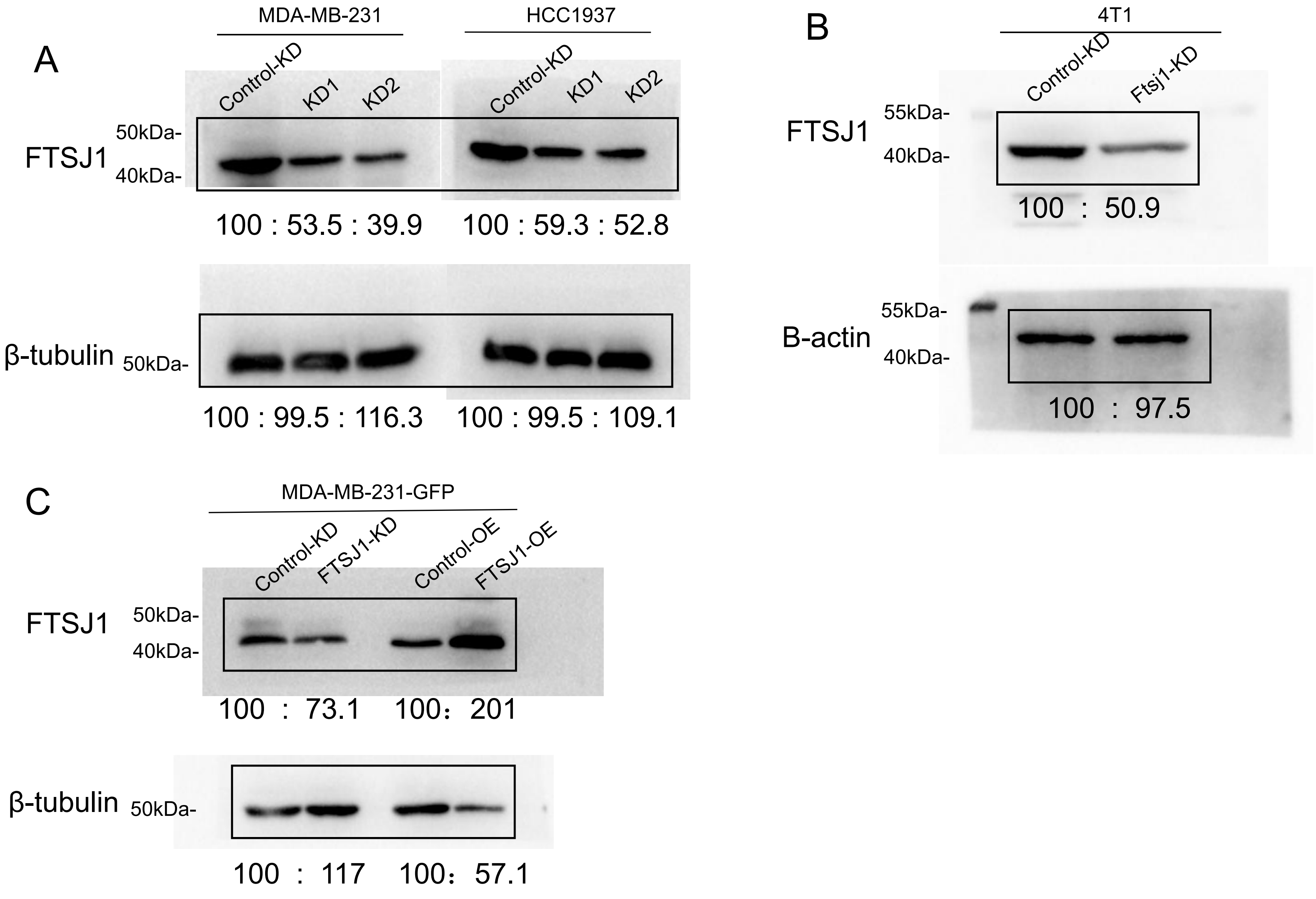

Figure S1.Knockdown efficiency in FTSJ1-KD and control (A) MDA-MB-231 and HCC1937 cells, (B) 4T1 cells, include densitometry readings/intensity ratio.(C)Knockdown and overexpression efficiency in FTSJ1-KD, OE and control MDA-MB-231-GFP cells, include densitometry readings/intensity ratio.
